# Interplay between Metabolism Reprogramming and Epithelial-to-Mesenchymal Transition in Cancer Stem Cells

**DOI:** 10.3390/cancers13081973

**Published:** 2021-04-20

**Authors:** Yoann Daniel, Elise Lelou, Caroline Aninat, Anne Corlu, Florian Cabillic

**Affiliations:** 1 INSERM, Université Rennes, INRAE, Institut NuMeCan (Nutrition, Metabolisms and Cancer), F-35000 Rennes, France; yoann.daniel@univ-rennes1.fr (Y.D.); elise.lelou@univ-rennes1.fr (E.L.); caroline.aninat@univ-rennes1.fr (C.A.); florian.cabillic@univ-rennes1.fr (F.C.); 2 Laboratoire de Cytogénétique et Biologie Cellulaire, CHU Rennes, F-35000 Rennes, France

**Keywords:** cancer stem cell, cell plasticity, metabolism reprogramming, epithelial-to-mesenchymal transition, catecholamines

## Abstract

**Simple Summary:**

Tumor cells display important plasticity potential. Notably, tumor cells have the ability to change toward immature cells called cancer stem cells under the influence of the tumor environment. Importantly, cancer stem cells are a small subset of relatively quiescent cells that, unlike rapidly dividing differentiated tumor cells, escape standard chemotherapies, causing relapse or recurrence of cancer. Interestingly, these cells adopt a specific metabolism. Most often, they mainly rely on glucose uptake and metabolism to sustain their energy needs. This metabolic reprogramming is set off by environmental factors such as pro-inflammatory signals or catecholamine hormones (epinephrine, norepinephrine). A better understanding of this process could provide opportunities to kill cancer stem cells. Indeed, it would become possible to develop drugs that act specifically on metabolic pathways used by these cells. These new drugs could be used to strengthen the effects of current chemotherapies and overcome cancers with poor prognoses.

**Abstract:**

Tumor cells display important plasticity potential, which contributes to intratumoral heterogeneity. Notably, tumor cells have the ability to retrodifferentiate toward immature states under the influence of their microenvironment. Importantly, this phenotypical conversion is paralleled by a metabolic rewiring, and according to the metabostemness theory, metabolic reprogramming represents the first step of epithelial-to-mesenchymal transition (EMT) and acquisition of stemness features. Most cancer stem cells (CSC) adopt a glycolytic phenotype even though cells retain functional mitochondria. Such adaptation is suggested to reduce the production of reactive oxygen species (ROS), protecting CSC from detrimental effects of ROS. CSC may also rely on glutaminolysis or fatty acid metabolism to sustain their energy needs. Besides pro-inflammatory cytokines that are well-known to initiate the retrodifferentiation process, the release of catecholamines in the microenvironment of the tumor can modulate both EMT and metabolic changes in cancer cells through the activation of EMT transcription factors (ZEB1, Snail, or Slug (SNAI2)). Importantly, the acquisition of stem cell properties favors the resistance to standard care chemotherapies. Hence, a better understanding of this process could pave the way for the development of therapies targeting CSC metabolism, providing new strategies to eradicate the whole tumor mass in cancers with unmet needs.

## 1. Introduction

In the 1920s, Warburg first claimed that tumor cells displayed metabolic dysregulation. Based on studies that showed superior lactate production in tumors compared to non-tumor cells, he postulated that “the respiration of all tumor cells is damaged” [1,2]. Since then, the landscape of metabolic reprogramming in tumor cells has become more complex. Studies have shown that tumor cells may use glycolysis, glutaminolysis, or lipid catabolism to meet their energy needs and maintain their anabolism. In addition, inconsistent with Warburg’s theory, they have shown that most tumor cells retain functional mitochondria [3,4,5,6] and revealed an unexpected inter- and intra-tumoral metabolic heterogeneity [7].

**Figure 1:**
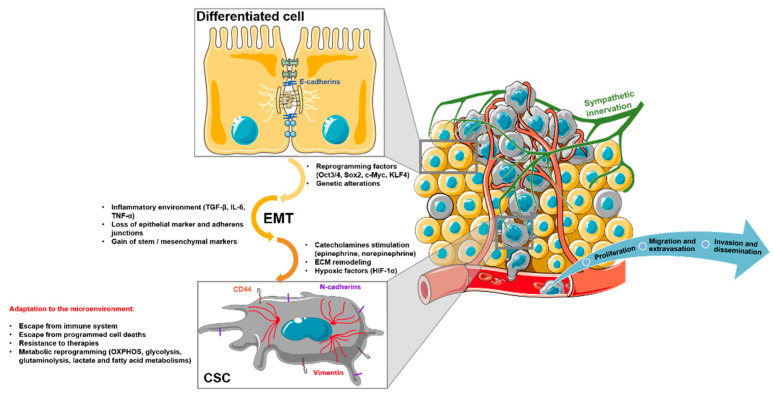
Tumor microenvironment drives epithelial-to-mesenchymal transition and tumor progression. Under the effects of genetic alterations and/or the influence of the microenvironment (inflammatory cytokines, hypoxic factors, catecholamines), differentiated tumor cells can undergo epithelial-to-mesenchymal transition and metabolism reprogramming. These alterations lead to the development of cancer stem cells that escape programmed cell death, evade the host immune system, and resist standard care chemotherapies. ECM: extracellular matrix; CSC: cancer stem cell; EMT: epithelial-to-mesenchymal transition; OXPHOS: oxidative phosphorylation.

Increasing evidence shows that metabolic reprogramming in tumor cells partly relies on epithelial-to-mesenchymal transition (EMT, Figure 1). EMT is a biological process that takes place during embryonic development, tissue repair, and regeneration, or neoplastic transformation, by which epithelial cells lose cell polarity and cell-cell adhesion and gain migratory/invasion properties [8,9,10,11]. It is a multistep process orchestrated by EMT-inducing transcription factors (EMT-TFs). EMT ultimately gives rise to a fully mesenchymal cell state, passing through various intermediate cell types, the so-called quasi-mesenchymal cells, which can co-express epithelial (E-cadherin) and mesenchymal markers (N-cadherin, vimentin) [12]. Unlike normal cells, tumor cells usually undergo incomplete EMT, which endows cells with the ability to escape apoptosis, anoikis, and senescence and evade immune surveillance. Importantly, quasi-mesenchymal cells, rather than fully mesenchymal cells, were shown to efficiently seed metastases at distant sites, likely because they are able to carry out more easily the reverse mesenchymal-epithelial transition (MET) [13]. During the EMT process, tumor cells also acquire progenitor/stem cells (CSC) marker expression (e.g., CD44, CD133, Nanog, and OCT-4) and properties, notably self-renewal, asymmetric cell division, tumor initiation, drug resistance, and disease recurrence. CSC are in a dynamic state and display high plasticity potential. They can move from quiescent to proliferative states and/or commit to differentiation, the derived-differentiated cells being able to retrodifferentiate toward CSC state. This phenotypical conversion of tumor cells is accompanied by a metabolic rewiring that is likely to contribute to therapeutic escape [14]. Accordingly, the tumors affected by a high EMT level are poorly differentiated tumors with CSC enrichment, and their prognosis is poor [15,16,17].

Importantly, the inflammatory environment associated with some cancers has been shown to contribute to both EMT and establishment of a tissue niche that supports CSC genesis (Figure 1). We have previously shown that an inflammatory environment containing transforming growth factor (TGF)-β1, interleukin (IL)-6, and tumor necrosis factor (TNF)-α triggers EMT and commits differentiated tumoral hepatocytes into a retrodifferentiation program toward cells harboring a CSC phenotype [18,19]. This retrodifferentiation is associated with metabolic reprogramming, the acquisition of invasive properties, and resistance to chemotherapy [20]. Similarly, in breast cancer, IL-6 secreted by tumor cells participates in the initiation of EMT, leading to an enrichment in CSC-like CD44^+^ cell subpopulation, with mesenchymal phenotype and invasive properties [21,22]. TGF-β1/TNF-α/Interferon (IFN)-γ-dependent activation of the indoleamine 2,3 dioxygenase (IDO) pathway in lung, liver, and breast cancer cell lines also drives EMT and confers immunomodulatory properties to the cells, which both contribute to metastasis and cancer progression [23]. Besides proinflammatory cytokines, neurotransmitters contribute to EMT in well-vascularized and -innervated tumors [24,25,26,27,28]. Among them, the norepinephrine and epinephrine catecholamines have been shown to participate in EMT and metabolic reprogramming in liver and colorectal cancers [29,30]. In this review, we discuss the interplay between stemness acquisition and metabolism reprogramming, focusing on the role of the microenvironment, notably catecholamines.

## 2. Metabolism in Cancer Stem Cells

**Figure 2:**
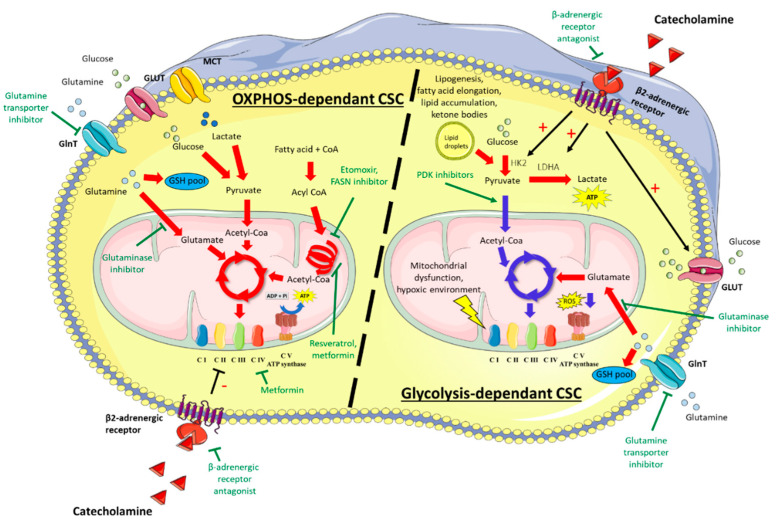
Glycolysis- or OXPHOS-dependent cancer stem cells. Depending on tissue location and quiescence/proliferation status, CSC preferentially use glycolysis or OXPHOS to meet their energy needs. Glycolysis-dependent CSC are characterized by increased lactate production and/or glutaminolysis. Catecholamines favor the glycolytic profile by increasing the expression of glucose transporters and key glycolytic enzymes (HK2, LDHA). Lower use of OXPHOS reduces the ROS production. In OXPHOS-dependent CSC, the TCA cycle is fed from glucose, glutamine, and fatty acids. Metabolic-targeted therapies under investigation are indicated in green. Red arrows indicate intensified signaling pathways and blue arrows indicate reduced signaling pathways. CSC: cancer stem cell; OXPHOS: oxidative phosphorylation, ROS: reactive oxygen species; HK2: hexokinase 2; LDHA: lactate dehydrogenase A; GSH: glutathione; GLUT, glucose transporter; GlnT, glutamine transporter; MCT, monocarboxylate transporter; CI: complex I of the respiratory chain.

Metabolic reprogramming is widely described in various biological processes such as inflammation, immune response, and development [31]. For instance, reprogramming from glycolysis to oxidative phosphorylation (OXPHOS) takes place during the differentiation of embryonic stem cells [32,33,34] while the reverse shift, from OXHPOS to glycolysis, occurs during genetic reprogramming of somatic cells into induced pluripotent stem cells (iPS) [35,36]. Unfortunately, the high plasticity of tumor cells allows them to move along metabolic/differentiation axes [37] to adapt to their environment. This property drives chemoresistance and cancer recurrence. Importantly, reports in the literature suggest a tissue-dependent heterogeneity of CSC: some are glycolysis-dependent, others preferentially use OXPHOS, and still others display hybrid phenotypes [38,39,40,41] (Figure 2).

Like differentiated tumor cells, CSC can get their energy from glucose [16,42,43,44,45]. Adoption of a glycolytic metabolism can be driven by microenvironmental constraints, such as a hypoxic environment, or induced by mitochondria dysfunctions [46,47,48,49]. For example, mutations in genes encoding subunits of the respiratory chain complexes have been associated with the transformation of differentiated tumor cells into CSC [50]. Recently, it has become widely accepted that adoption of a glycolytic metabolism can be an unforced event which may occur even when oxygen is available in the microenvironment and in cells lacking mitochondria dysfunctions. As a result of glycolytic reprogramming, the glycolysis intermediate pyruvate is converted to lactate instead of feeding the tricarboxylic acid (TCA) cycle [17,35,41,51,52]. The lactate synthesis yields less ATP per glucose than OXPHOS, but this is offset by both increased glucose uptake and lactate dehydrogenase A (LDHA) activity [17,39]. Importantly, this reprogramming allows cells to sustain their anabolism while restricting the production of reactive oxygen species (ROS) that are highly toxic for CSC endowed with low levels of ROS detoxification enzymes [53,54] (Figure 2). Consistently, dual inhibition of LDHA, which blocks glycolysis end-step, and pyruvate dehydrogenase kinase 4 (PDK4), which favors the flow of pyruvate into the TCA cycle, represses the CSC phenotype in CD133^+^/PLC/PRF/5 hepatocellular carcinoma (HCC) cells [55]. Interestingly, our team has recently shown that selective inhibition of PDK4 by dichloroacetate or shRNA in hepatic CSC succeeded to reverse the resistance to sorafenib [20].

Besides glucose, amino acids, fatty acids (FA), and extracellular catabolites can support the metabolism of CSC. Glutaminolysis has an important role in carcinogenesis and some KRAS-driven non-small cell lung cancers (NSCLC), among cancers with the worse outcome, display glutaminolysis dependency [56]. Apart from the production of energy through the TCA cycle via α-ketoglutarate intermediate, glutaminolysis is involved in the synthesis of amino acids, nucleotides, and FA [57]. Moreover, glutaminolysis has beneficial effects on the redox balance by generating antioxidant molecules such as glutathione (GSH), which interacts with O_2_^-^ produced by the electron transport chain (Figure 2). This antioxidant property is illustrated by the reduction of ROS levels in neonatal hepatocytes following glutamine supplementation and explains, at least in part, why CSC particularly benefit from the use of glutaminolysis [58]. Accordingly, CSC in glioblastoma, pancreatic ductal adenocarcinoma, and B-cell acute lymphoblastic leukemia express CD9 that favors the localization of the glutamine transporter at the plasma membrane, thus promoting glutaminolysis [59]. Likewise, the inhibition of glutamine metabolism has been shown to attenuate stemness properties in hepatocellular carcinoma and pancreatic cancers [60,61].

FA provide another fuel pathway for CSC. The expression of FA translocase (a.k.a. CD36), which allows lipid uptake, is correlated with poor prognosis in various cancers [62], and CD36 is suggested as a stem cell marker in breast, brain, and adipose tissue tumors [63,64,65]. Elevated FA oxidation (FAO) is proposed to maintain CSC self-renewal by modulating lipid and membrane synthesis, quenching ROS through NADPH production, and promoting chemoresistance [66,67]. Pastò et al. reported an increased transcription level of FAO-related genes in CSC from epithelial ovarian cancer [68]. Chen et al. also reported increased FAO, associated with a decrease of FA elongation, in tumor-initiating cells (TIC) from HCC cell lines that overexpress the stem cell marker Nanog [69]. On the other hand, a lipogenic switch is proposed to favor the production of monounsaturated lipids that are less susceptible to lipid peroxidation, restricting the detrimental effect of ROS in CSC [70]. In addition, the excess of free FA can be converted to triglycerides and stored as lipid droplets to avoid toxicity. Accumulation of such lipid droplets is frequently found in CSC [20,71]. Of note, the lipogenic switch can strengthen the glycolytic reprogramming, as FA synthesis allows the production of the NAD^+^ needed for glycolysis. Accordingly, Vazquez-Martin et al. demonstrated that increased FA synthesis and neutral lipid accumulation are associated with glycolysis reprogramming in iPS and CSC [70] (Figure 2).

The self-degradation process of unnecessary macromolecules or damaged organelles, called autophagy, also allows cells to maintain cellular homeostasis and adapt their metabolism to overcome stressful conditions. Notably, in nutrient-poor environments, Mit/TFE family of transcription factors regulate the transcription of genes involved in the lysosomal-autophagy pathway and in lipid metabolism (lipophagy, β-oxidation, and ketogenesis), such as PPARα (peroxisome proliferator-activated receptor-α) and PGC1α (PPARγ co-activator 1α). Autophagy is especially relevant for CSC, as they primarily reside in nutrient-poor and acidic environments known to hold pro-autophagic signals [72]. Lipid droplets, typically enriched in CSC, can be engulfed in autophagosomes and targeted to lysosomes, in which acid hydrolases generate simple molecules to sustain core anabolic functions. Moreover, mitophagy of senescent mitochondria is useful to reduce the level of ROS, protecting CSC from DNA damages. Autophagy has been shown to support the maintenance of hepatic CSC by removing mitochondria-associated P53, preventing P53 binding on Nanog promoter that results in the suppression of Nanog expression [73]. Likewise, autophagy supports the maintenance of CD44^+^CD24^−/low^ breast CSC via IL-6-mediated autocrine signaling [74,75].

Finally, extracellular catabolites produced by the cells surrounding the tumor, such as ketone bodies and lactate, represent another unexpected and important source of energy for CSC [76,77]. Ketone bodies have been shown to increase the expression of stemness-related genes in breast cancer [78,79]. In addition, yet long regarded as a dead-end waste product, lactate can be selectively up-taken by CSC and stimulate stem cell growth [80]. In patient-derived colorectal cancer organoids, Zhao et al. proposed a metabolic commensalism based on the exchange of lactate between CSC and non-CSC. Lactate secreted by glycolytic non-CSC in the environment through the monocarbohydrate transporter MCT4 is taken up by CSC through MCT1, enhancing OXPHOS activity and promoting self-renewal of CSC via ROS/AKT/Wnt/β-catenin pathway [81]. Consistent with these observations, silencing MCT transporters in either CSC or non-CSC reduces the tumor-initiating activity. Notably, oxidative tumor cells can force the surrounding stromal cells to adopt a glycolytic metabolism in order to obtain additional lactate for their own metabolism [82]. The underlying mechanism of action of lactate is starting to be elucidated. In glycolytic cancer cells, lactate activates an NDRG3/Raf/ERK tumor-growth pathway whereas it promotes mitochondrial biogenesis, pro-angiogenic signaling, and glutaminolysis in oxidative cancer cells [83]. In addition, lactate, by scavenging free radicals, also prevents the initiation step of lipid peroxidation [84].

Of note, the glycolytic shift toward lactate production does not preclude the course of OXPHOS, which can be activated by alternative fuels such as FA, amino-acids, or extracellular catabolites (Figure 2). Accordingly, various studies have shown that OXPHOS could be efficient in CSC, some of which preferentially adopt a mitochondrial oxidative metabolism [85,86]. The significant contribution of OXPHOS in cancer stemness has notably been proven from the high level of mitochondria biogenesis driven by PPAR-γ co-activator 1a (PGC-1a) in stem-like cancer cells [40], the up-regulation of enzymes involved in the respiratory chain in ovarian CSC [68], and the OXPHOS-dependency of CSC in small cell lung cancer [87,88]. Importantly, the presence of functional mitochondria is useful to sustain increased energy needs when CSC move from quiescent to a proliferative state or commit to a differentiated state.

## 3. Metabolism Reprogramming, a First Step for EMT?

Renewed interest in cancer cell metabolism has raised questions about the interplay between metabolism reprogramming and EMT, while it is not easy to formally determine which one governs the other.

Some well-known EMT-TFs, tumor suppressor genes and oncogenes, have been described to rewire the cell metabolism. Snail (a.k.a. SNAI1) regulates many genes involved in glucose metabolism, such as glucose phosphate isomerase and aldolase [89]. More recently, Snail has been shown to promote glucose metabolism by inducing epigenetic silencing of FBP1 in basal-like breast cancer [90]. Likewise, Snail limits OXPHOS by repressing the activity of both cytochrome C oxidase and mitochondrial respiratory complex I. Moreover, Snail reprograms glucose metabolism toward the pentose phosphate pathway by repressing phosphofructokinase platelet (PFKP), a rate-limiting enzyme of glycolysis [91]. Zeb1, another EMT-TFs, also contributes to metabolic plasticity in pancreatic tumor cells, allowing cells to switch between OXPHOS or glycolytic profile: whichever is most appropriate [92]. The third example of the role of EMT-TFs in the metabolism switch, Twist, has been shown to decrease mitochondrial mass while increasing glucose consumption and lactate production in twist-positive breast cancer cells. This energy metabolic reprogramming is induced via the activation of β1-integrin, FAK, and PI3K/AKT/mTOR pathway and the inhibition of p53 pathway [93]. Of note, PI3K activation was shown to directly coordinate glycolysis by mobilization of aldolase from actin [94]. In addition, loss of the tumor suppressor p53, which is best known to contribute to the acquisition of stemness features [95], was shown to activate the phosphoglycerate mutase, a rate-limiting enzyme of glycolysis, and downregulate the electron transport activity [96,97]. Finally, KRAS, a well-known oncogene, which up-regulates the transcription of the major EMT-promoting factor Myc, also increases glycolysis by enhancing glucose uptake through the glucose transporter GLUT1 in both lung and colorectal carcinomas [98,99]. It was also shown to increase glycolysis by inhibiting glutaminolysis in pancreatic cancers [100].

Conversely, metabolic reprogramming may cause EMT and stemness. In 2011, Folmes et al. demonstrated that glycolytic gene expression precedes the expression of genes associated with pluripotency, suggesting that a glycolytic switch may induce cell dedifferentiation in mouse embryonic fibroblasts [101]. The causative role of glycolysis reprogramming in EMT is strengthened by data in ovarian cancers. Indeed, Siu et al. showed that IL6-dependent overexpression of hexokinase II (HK2), the rate-limiting first step of glycolysis, results in the expression of stem markers (Nanog, Oct4, Klf4, Sox9, and CD117) and increases invasiveness, subsequent to FAK-ERK-MMP9 signaling pathway activation [102]. LDHA that catalyzes the subsequent step of glycolysis, the conversion of pyruvate into lactate, promotes the transcription of genes involved in the EMT process by increasing H3K27 acetylation [103]. Hence, epithelial marker E-cadherin is down-regulated whereas EMT-TFs, mesenchymal markers, and TGF-βR1 were found positively regulated in papillary thyroid carcinoma [103]. Increased lactate levels in the microenvironment have also been shown to induce EMT in breast cancers [44], and treatment of breast cancer cells with lactate leads to gene signature associated with stemness [78]. In addition, the supplementation of the culture medium with lactate and high glucose concentration induces the expression of genes related to stemness (Nanog, Oct4, and Sox2) as well as the resistance to anoikis in HCC CD133^-^/PLC/PRF/5 cell line [55]. Recently, Sandforth et al. reinforced the role of lactate production and of the resulting acidic microenvironment in the acquisition of stemness features. They showed that increased lactate uptake following the overexpression of the membrane transporter monocarboxylate transporter-1 (MCT1) leads to the acquisition of stem markers (Nestin, Oct4, Klf4, Nanog) by pancreatic adenocarcinoma cells [104]. As regards the mode of action, it was shown that acidic pH promotes autocrine TGFβ2 signaling, which induces both partial EMT and accumulation of triglycerides in lipid droplets that represent energy stores available for FA oxidation and ATP needs [105].

Besides glycolytic switch, alterations in the lipid metabolism have also been shown to promote EMT and stemness in various cancers [106,107,108,109]. Increased lipid content is associated with EMT in breast cancers and lipid signatures are useful to segregate EMT and non-EMT tissues [110]. In addition, elevated expression of CD36 promotes EMT through free FA uptake and subsequent activation of Wnt/TFGβ signaling in hepatocellular carcinoma [111]. Likewise, Wang et al. recently demonstrated that CD36 upregulates the DEK proto-oncogene transcription and promotes migration and invasion via GSK-3β/β-catenin-mediated EMT in gastric cancer [112]. Also, the overexpression of FABP4, a FA transport protein, upregulates the EMT-TF Snail and the matrix-metalloproteinases MMP2/9. It promotes both metabolism and EMT, partly by activating Akt pathway in colon cancer cells [113]. In addition, FA synthase, a key enzyme of *de novo* lipogenesis that converts dietary carbohydrates to fatty acids, mediates EMT in breast cancer cells, possibly through regulating liver fatty acid-binding protein (L-FABP) and VEGF/VEGFR-2 [107,114]. Accordingly, inhibition of FA synthase has been shown to attenuate CD44-associated signaling and reduce metastases in colorectal cancer [106]. Of note, reduced expression of CD44 attenuates the activation of MET, Akt, FAK, and paxillin, all known to regulate adhesion, migration, and invasion. At last, metabolic reprogramming toward increased mevalonate pathway activity that produces cholesterol but also prenyl anchors is associated with EMT and cell retrodifferentiation toward CSC [108]. Myc-driven mevalonate metabolism has recently been shown to maintain brain tumor-initiating cells and immature sphere-forming cells [115,116].

Interestingly, autophagy and EMT share signaling pathways (TFG-β, STAT3) and are linked in an intricate relationship, notably through reciprocal interaction between cytoskeleton and mitochondria [117,118]. Autophagy is useful for the EMT process as it helps cells to overcome anoikis after loss of cell anchorage and to escape immune surveillance during cell spreading [119]. Upregulation of autophagy by HIF-1α favors EMT and metastatic ability of CD133+ pancreatic cancer stem-like cells. Likewise, DNA-damage regulated autophagy modulator 1 (DRAM1), known as a target of TP53-mediated autophagy, has been reported to promote migration and invasion abilities of glioma stem cells [120]. However, autophagy could also ensure the degradation of specific EMT-TF, e.g., Snail and Twist [121,122], and favors mitochondrial fusion and the reconstitution of the mitochondrial network. This limits the accumulation of free mitochondria below the cell membrane, preventing actin skeleton remodeling and the subsequent formation of filopodia and lamellipodia that support cell motility and migration [117]. Accordingly, autophagy inhibition reduces the incidence of pulmonary metastases in an orthotopic mouse model of HCC [123] and potentiates the anti-EMT effects (migration/invasion) of alterolol in melanoma cells [124]. Likewise, drugs with pro-autophagic properties (e.g., alisertib, an aurora kinase A inhibitor) inhibit EMT in human ovarian and colorectal cancer cells [125,126].

Finally, mitochondrial dysfunctions may also cause EMT and stemness. Mutations in the succinate dehydrogenase (SDH) B subunit gene, involved in the complex II of the respiratory chain and the TCA cycle, are associated with the induction of the EMT-TFs, TWIST1, and Snail [127,128,129]. Moreover, knockout of the mitochondrial pyruvate carrier 1 (MPC1), which restricts the entry of pyruvate in the TCA cycle, leads to increased expression of the stem cell marker Nanog [130]. Conversely, the expression of mitochondrial intermembrane space import and assembly protein 40 (CHCHD4), which increases glutamine metabolism and respiratory chain activity, reduces the expression of EMT-related genes [131].

## 4. Catecholamines: Actors of the Interplay between EMT and Metabolism?

In recent years, increasing reports have put forth the link between cancer development and catecholamines, especially epinephrine and norepinephrine, which emerged as new actors able to influence the growth and aggressiveness of many types of tumors. Indeed, catecholamines have been shown to modulate both EMT and metabolic changes in cancer cells and chronic stress to trigger a CSC phenotype in tumor cells in immunodeficient mice models [132]. Interestingly, chronic stress is characterized by a release of epinephrine in the bloodstream by the adrenal gland and an increase of sympathetic nerve activity [133]. Thus, pro-tumoral effects of catecholamines are observed both in animal cancer models exposed to chronic stress [134,135] and in clinical data. Moreover, high concentration of epinephrine in patient blood samples is correlated with a lower median survival rate in pancreatic [136], breast [132], and hepatocellular cancers [137].

On a molecular level, catecholamines can promote EMT through the activation of EMT-TFs such as ZEB1, Snail, or Slug (SNAI2). In the gastric cancer cell lines MGC-803 and HGC-27, isoproterenol, a β_2_-adrenergic receptor (β_2_-ADR) agonist, activates ZEB1 and Snail, leading to the expression of EMT (CDH2, Vimentin, α-SMA) and CSC (CD44) markers through the STAT3 signaling pathway activation [138]. Similarly, in the gastric adenocarcinoma cell lines BGC-823 and SGC-7901, norepinephrine represses E-cadherin and induces vimentin expressions through the activation of the β_2_-ADR-HIF1α-Snail axis [139]. Moreover, Slug can be induced by norepinephrine in the ovarian cancer cell lines SKOV-3 and PA-1 [140]. This induction is mediated by a β_2_-ADR-HIF1α and c-Myc dependent pathway, which results in the induction of hTERT. Therefore, hTERT activates NF-κB, leading to an increased expression of Slug, enhancing the EMT process. In addition, catecholamines favor the secretion of extracellular matrix (ECM) remodeling enzymes by the cancer cells, enhancing their invasion capacities. Li et al. reported that the activation by catecholamines of both α_1_- and β_2_-ADR leads to the release of matrix metalloproteinase 7 (MMP-7) and ADAM12, which in turn allows the EGFR transactivation in HCC cells and their invasiveness [141]. In the ovarian cancer cell lines EG and SKOV3, as well as in the colorectal SW480 cancer cell line and in oral squamous cell carcinoma (OSCC) SCC25 and Cal27 cell lines, catecholamines by a β-ADR-depending pathway increase the cell migration and invasion by up-regulating MMP-2 and MMP-9 [142,143,144,145]. In the OSCC cell lines SCC25 and Cal27, norepinephrine stimulation is also associated with the enhancement of CSC-like phenotype, notably by an induction of the expression of stem markers (CD44, OCT4, SOX2, and ALDH1) and of the formation of colonies and spheres [146]. Moreover, catecholamines can also promote the EMT by enhancing the production of TGF-β. For example, in the colorectal HT29 and lung A549 cancer cell lines, activation of β_2_-ADR leads to an induction of TGF-β1 by a cAMP-dependent pathway. This production enables the activation of the TGF-β receptor I (TGF-βRI) and the induction of Snail by both a stabilization of HIF-1α and a phosphorylation of Smad3 [146]. Epinephrine is also able to induce TGF-β1 in the pancreatic cancer cell line PANC-1 [136]. In these cells, epinephrine induces the translocation of the RNA binding protein human antigen R (HuR) in the cytoplasm as well as its phosphorylation on S221. This translocation leads to an activation of the TGF-β pathway. One hypothesis suggested by the authors to explain the induction of TGF-β1 in their model is that HuR may allow the stabilization of its mRNA. In both these publications, the effects of catecholamines result in an enhancement of the migration, the E-cadherin repression, and the vimentin induction. Norepinephrine can also indirectly enhance EMT (N-cadherin, Vimentin, and Snail) and stemness (Nanog) markers in HCC cells by activating hepatic stellate cells that release the secreted frizzled-related protein 1 (sFRP1), which leads to the activation of Wnt/β-catenin pathway in the tumor cells, thus favoring HCC progression [147]. Last but not least, catecholamines are also able to enhance the production of pro-inflammatory cytokines, which are involved in EMT [18,21,22,23,148]. Epinephrine induces IL-6 in the HCC HepaRG cell line [149] whereas β-ADR activation by norepinephrine mediates its induction in the ovarian cancer lines SKOV3.ip1, Hey-A8, and EG [150] as well as in the melanoma cell lines A375 and Hs29-4T [151]. In addition to IL-6 secretion, metastatic melanoma cells can release IL-8 and VEGF under a β_2_-ADR-dependent catecholamines stimulation [152]. This β_2_-ADR-dependent VEGF secretion also promotes angiogenesis in gastric cancer cells [153].

Importantly, catecholamines can also dysregulate some metabolic pathways in cancer cells. Cui et al. recently found, in breast cancer models, that epinephrine is able to induce stem-like cell phenotype and stem cell factor expression through an increase of the glycolysis [132]. They showed that epinephrine, by activating the β_2_-ADR, induces the expression of LDHA, resulting in lactate accumulation and a cytosolic acidification to a pH 6.4. This weak acidic environment allows the stabilization of the proto-oncogene c-Myc by its interaction with the deubiquitinase USP28. Hence, c-Myc promotes the induction of Slug, a well-known regulator of EMT and stemness in cancer cells. Furthermore, they showed that a high serum epinephrine concentration is correlated with a higher level of LDHA, USP28, c-Myc, and Slug in the tumoral tissues of 83 breast cancers. Overall survival and disease-free survival rates are also better in patients with a low serum epinephrine concentration. Moreover, in a diethylnitrosamine (DEN)-induced HCC mouse model, epinephrine, by a β_2_-ADR activation, promotes the glucose metabolism but also the OXPHOS, favoring the tumor growth [154]. Indeed, by inhibiting the autophagy through an Akt pathway, a stabilization of HIF1α is observed, which then induces further genes involved in glucose metabolism such as the GLUT1 and the HK2. This mechanism participates in sorafenib chemoresistance in this model. HK2 is also found overexpressed in HCC tissues. This overexpression was positively correlated to the ADRB2 overexpression in these tissues, and both were associated with poor prognosis [155]. In fact, Kang et al. demonstrated in the 4T1 mouse breast cancer cell line that HK2 expression is regulated by the activation of the adrenoceptor, thus regulating glycolysis metabolism [156]. In addition, a new HCC classification based on metabolism-related genes from transcriptomic data showed that the HCC C1 subclass, categorized with a high metabolic gene expression profile including amino-acid or lipid metabolisms signatures and partly with a positive EMT signature profile, is enriched in “Dopamine/Epinephrine/Norepinephrine biosynthesis” signatures [157]. These results reinforce the role of catecholamines in the interplay between EMT and metabolisms in some tumor cells.

In conclusion, the release of catecholamines in the microenvironment of the tumor can facilitate several pro-tumoral actions related to EMT and metabolism reprogramming, especially glycolysis and OXPHOS, as well as the acquisition by the cancer cell of stemness properties, altogether leading to treatment resistance. The fact that most of these effects are β_2_-ADR-dependent suggests that antagonist of this receptor could represent an adjuvant treatment of cancer therapy in order to slow down tumor aggressiveness.

## 5. Conclusions/Perspectives

CSC are likely to cause chemotherapy resistance and recurrence of cancers. Implementing therapeutic approaches that achieve efficient removal of CSC is thus crucial for defeating cancer. However, CSC share numerous properties with normal stem cells, including expression of cell surface markers (CD44, CD133) and activation of signaling pathways (Wnt, Notch), making it difficult to develop therapies targeting CSC while sparing normal stem cells [79,158]. Since 2010, cell metabolism has become an accepted hallmark of cancer. Accumulative data support the metabostemness theory, which suggests that metabolic reprogramming favors EMT and stemness, resulting in pejorative outcomes for patients. Questions then arose as to whether CSC adopt a specific metabolism, providing opportunities for targeted therapies. The scientific literature reports tissue-dependent metabolic heterogeneity and flexibility, which allows CSC to rewire their metabolism depending on microenvironmental constraints. Lack of uniformity in the metabolism of CSC complicates therapeutic developments. Nevertheless, among various options, glutaminolysis-dependency or extensive use of autophagy may provide attractive targets. In recent years, various drug candidates targeting metabolism, some of which are likely relevant for CSC, have entered clinical investigation, including autophagy activators (e.g., metformin, alisertib) [126], PDK inhibitors [159], glutamine transporter inhibitors and glutaminase inhibitors (e.g., telaglenastat) [61,160,161], FA synthase inhibitors [162,163], P53 activators [164,165], and β-adrenergic receptor antagonists [166,167]. Further studies are warranted to determine the efficacy of these drugs in combination with standard care chemotherapies or synthetic lethal approaches in cancers with unmet needs.

## Data Availability

No new data were created or analyzed in this study. Data sharing is not applicable to this article.
